# Effect of maternal supplement beverage with and without probiotics during pregnancy and lactation on maternal and infant health: a randomized controlled trial in the Philippines

**DOI:** 10.1186/s12884-018-1828-8

**Published:** 2018-05-31

**Authors:** Jacinto Mantaring, Jalil Benyacoub, Raul Destura, Sophie Pecquet, Karine Vidal, Sheri Volger, Valerie Guinto

**Affiliations:** 1 Department of Pediatrics, Philippines General Hospital, City, Manila, Philippines; 2 Institute of Nutritional Science, Nestlé Research Center, Vers-chez-les-Blanc, 1000, 26 Lausanne, Switzerland; 3 Internal Medicine & Infectious Diseases, Philippines General Hospital, Manila, Philippines; 4 Clinical Research, Nestlé Nutrition, Vevey, Switzerland; 5 Janssen R&D LLC, Welsh & McKean Roads, Springhouse, PA 19477 USA; 6 Department of Obstetrics & Gynecology, Philippines General Hospital, Manila, Philippines

**Keywords:** Maternal supplementation, Probiotics, *Lactobacillus rhamnosus*, *Bifidobacterium lactis*, Diarrhea, Safety, Infant growth

## Abstract

**Background:**

Adequate nutrition is essential during pregnancy and lactation to provide sufficient energy and nutrients to meet the nutritional requirements of the mother, fetus and infant. The primary objective of this study was to assess the effect of a maternal nutritional supplement enriched with probiotics during pregnancy and early lactation on the incidence of infant diarrhea.

**Methods:**

Healthy, pregnant (24–28 weeks gestation) women were randomized 1:1:1 to receive either no supplement or two servings per day of an oral supplement (140 kcal/serving) providing 7.9 g protein, multivitamin/minerals, and enriched or not with the probiotics *Lactobacillus rhamnosus* and *Bifidobacterium lactis*, from the third trimester of pregnancy until at least 2 months post-delivery. Incidence of infant diarrhea until 12 months post-delivery was analyzed by Poisson regression. The effect on maternal health, fetal growth, and infant growth and morbidity were also evaluated and analyzed by ANOVA.

**Results:**

A total of 208 mother/infant pairs were included in the analysis. No significant difference in the incidence of infant diarrhea was observed between the three study groups. The mean maternal weight gains at delivery were similar among groups, despite an increase in caloric intake in the supplemented groups. No statistically significant differences between groups were observed in incidence of pregnancy-related or fetal adverse outcomes. Mean weight-, length-, BMI- and head circumference-for-age z-scores were below the WHO median value for all groups. Post-hoc analysis to compare the effect of the combined supplement groups versus the no supplement group on infant growth parameters showed, at 12 months, that the combined supplemented group had gained statistically significant more weight (8.97 vs. 8.61 kg, *p* = 0.001) and height (74.2 vs. 73.4 cm, *p* = 0.031), and had a higher weight-for-age z-score (− 0.62 vs. -0.88, *p* = 0.045) than the no supplement group.

**Conclusions:**

Maternal nutritional supplement with or without probiotics given during late pregnancy and early lactation was well tolerated and safe. Even though no difference in incidence of infant diarrhea was observed between the three groups, the analysis of the combined supplemented groups showed beneficial effects of maternal supplementation on infant weight and length gains at 12 months.

**Trial registration:**

ClinicalTrial.gov: NCT01073033. Registered 17.02.2010.

## Background

There is a substantial body of evidence showing that maternal diet throughout pregnancy and during breastfeeding could influence the infant growth, development and health [1, 2]. Only a modest increase in energy intake is required during pregnancy compared to pre-pregnancy requirements. Indeed, approximately 100 and 300 additional kcal per day are needed in the first and second/third trimesters, respectively, with increased requirements during breastfeeding of 450 kcal per day [3]. Conversely, the recommended intake during pregnancy for several nutrients such as iron, folate and vitamin B6 are up to 50% higher than those for a non-pregnant state, which could prove challenging [3]. Geographic and sociodemographic differences have been observed regarding nutrient deficiencies in pregnancy. The World Health Organization (WHO) has warned that some nutrient deficiencies (especially for vitamin A, iodine, vitamin D, calcium and zinc) are more common in pregnant women in low resource countries compared to those with higher resources. Other micronutrients (i.e., vitamin B12, iron, and folate) are commonly deficient worldwide [3]. This is of particular importance considering that maternal undernutrition can lead to miscarriage and major developmental issues such as intrauterine growth restriction, low birth weight and premature births [4, 5]. As many as 800,000 neonatal deaths per year are from small for gestational age births, many of which are attributed to maternal undernutrition [6]. It was estimated that stunting, wasting, and micronutrient deficiencies account for almost 3.1 million child deaths annually [6]. Ultimately, maternal nutrition, especially micronutrient intake, is an important determinant of health as an adult. This has been shown to have an impact on the development of cardiovascular diseases and obesity [7, 8].

In developing countries, with a high risk of under nutrition, the use of nutritional supplements during pregnancy is encouraged to ensure adequate supply of nutrients for both mother and fetus [5]. In a recent Cochrane review, Haider and Bhutta evaluated the benefits of oral multiple-micronutrient supplementation during pregnancy on maternal, fetal and infant health outcomes. The analysis of 15 clinical trials conducted in low and middle-income countries revealed that multiple-micronutrient supplementation with iron and folic acid resulted in a significant decrease in the number of low birth weight or small-for-gestational age newborn infants, and a reduced rate of stillbirth [9].

Dramatic immune and physiological changes take place during pregnancy to accommodate the growing fetus; these include alterations in the gut and vaginal microbiome populations [10]. These changes may influence the maternal metabolic profile and may contribute to the metabolic and immunological health of the infant [10]. The composition of the microbiome is influenced by many factors, including diet and intake of nutritional bioactives such as probiotics [10]. Probiotics can regulate gut and vaginal microflora, and probiotic supplementation has been used in some trials as a strategy to influence pregnancy and infancy outcomes [10]. Maternal transfer of bacterial signals is possible during pregnancy and lactation [11]. This (as well as the mother’s diet and microbiota) can influence newborn microbial colonization and microbiota establishment which in turn may have an impact on infant’s short and long-term development parameters, including growth [11].

Prior to the initiation of our study, the safety and effect of a maternal nutritional supplement containing a mix of probiotics for pregnant and breastfeeding women on fetal and infant growth was only partially addressed. Safe use of probiotics has been documented. Indeed, infants born from mothers receiving *Lactobacillus rhamnosus* were shown to have a significantly reduced risk of developing atopic eczema during the first two [12] to seven [13] years of life and no adverse effects were reported [12, 13]. Researchers also observed an improved gut microbiota in babies born from women receiving probiotics during pregnancy [14] or during pregnancy and lactation [15], and again, these studies did not report any adverse events [14, 15]. Some probiotic strains have also been successfully used to improve the outcome of gastrointestinal diseases, in particular diarrhea [16, 17]. Diarrhea episodes are common infant infections and are a major health concern in pediatrics.

Among the probiotic strains, *Bifidobacterium lactis* and *Lactobacillus rhamnosus* have been reported to improve diarrheal outcomes in infants [18–23]. Therefore, we conducted this study to assess the effect of an oral maternal nutritional supplement formulated with these two probiotics on the incidence of diarrhea in infants from birth to one year, assessed as the primary endpoint of the trial. Secondary objectives included examining the effect of an oral maternal nutritional supplement on maternal health, fetal growth, and infant growth and morbidity up to one year of age. Due to the overall low incidence of diarrhea reported in this study, we conducted an additional post-hoc analysis. It evaluated the effect of the combined oral supplement groups (with and without probiotics) compared to the no supplement group on maternal health and fetal and infant growth.

## Methods

### Study design

A single center, randomized, double blind trial of three parallel-groups (supplement, supplement plus probiotics, and no supplement) was conducted at the Community Hospital in Muntinlupa City, Philippines, between April 2010 and September 2012. The study protocol was approved by the institution’s Ethics Committee (University of the Philippines Manila – National Institutes of Health Ethics Review Board; protocol number NIH 2009–027). The study was conducted in accordance with the Helsinki Declaration and complied with Good Clinical Practices as laid out in the International Conference on Harmonization guidelines. Written informed consent was obtained from all study participants after the nature and possible consequences of the study had been fully explained. The primary objective of the trial was to examine the effect of an oral nutritional supplement containing a mix of probiotics *Lactobacillus rhamnosus* and *Bifidobacterium lactis*, taken during pregnancy and up to minimally 2 months post-delivery on the incidence of diarrhea in infants from birth to one year of age. The secondary objectives were (i) in mothers, to assess the effect of oral nutritional supplements on fetal growth and on maternal health (weight gain and pregnancy-related adverse events [AEs]) during the third trimester of pregnancy and up to delivery; and (ii) in infants, to investigate the impact of nutritional supplement beverages on infant growth and morbidity.

### Study population

Healthy women at the beginning of the third trimester of pregnancy (24 to 28 weeks of gestation) who were willing to exclusively breastfeed for at least the first 2 months postpartum were recruited and enrolled in the study. The study protocol encouraged exclusive breastfeeding up to 6 months of age. The infants born to study participants were followed until one year of age. Pregnant women were excluded from the study if they had any of the following exclusion criteria: known allergy to cow’s milk, known history of lactose intolerance, previous diagnosis of human immunodeficiency virus (HIV) and hepatitis B, multiple pregnancy, high risk pregnancy (pre-eclampsia, diabetes, etc.), current or past participation in another clinical trial during the last three months, consumption of pro- and/or prebiotics-containing food/supplement in the month before inclusion.

### Study supplements and blinding

The supplement and supplement plus probiotics formulations consisted of proteins, carbohydrates, fats with vitamins and minerals in amounts intended for nutritional support during pregnancy. Each serving of 200 mL contained 140 kcal energy, 7.9 g of proteins, 21 g of carbohydrates, and 3.5 g of fat. It also contained minerals and vitamins (Table 1). The supplement plus probiotics contained the mix of probiotics composed of 7 × 10^8^ cfu of *Bifidobacterium lactis* CNCC I-3446 and 7 × 10^8^ cfu of *Lactobacillus rhamnosus* CGMCC 1.3724, per serving. Both supplements were manufactured by the sponsor, Nestlé (Product technology center, Konolfingen, Switzerland). The two supplements were indistinguishable and were supplied as powder, in sachets, with instructions to prepare 35 g of powder in 200 ml of water. Both products were blinded by the manufacturer. Thus, the identity of the specific products was blinded to the subjects, support staff and investigators. The reference group not taking the supplement was not blinded.

**Table 1:** Nutritional composition of the supplements

Energy, kcal	140
Fat, g	3.50
Linoleic acid, mg	73.2
Alpha-linoleic acid, mg	80.9
Docosahexaenoic acid, mg	43.8
Protein, g	7.9
Carbohydrates, g	21
Minerals, mg	
Sodium	87
Chloride	205
Calcium	254
Magnesium	32
Iron	7
Zinc	2.6
Vitamins	
Vitamin A, IU	438
Vitamin D, IU	35
Vitamin E, IU	4.60
Vitamin C, IU	19.6
Vitamin B1, μg	217
Vitamin B6, μg	263
Niacin, μg	3045
Folic acid, μg	125
Panthotenic acid, μg	1295
Biotin, μg	9.80

The composition of the supplement plus probiotics is identical to the supplement alone except for the addition of *L. rhamnosus* (7 × 10^8^ cfu) and *B. Lactis* (7 × 10^8^ cfu)

### Randomization

An in-house computer program (TrialSys) was used to generate a randomization sequence to allocate expecting mothers to either the supplement, supplement plus probiotics, or no supplement groups. Eligible pregnant women were randomized into the three groups with a 1:1:1 ratio. The investigator accessed allocation numbers via a web-based application. Random allocation remained concealed until assigned by computer program and could not be predicted based on previous assignments.

### Trial procedure

Each supplemented group received 2 × 200 ml serving per day of the allocated nutritional supplement beverage (to be taken in the morning and evening). The no supplement group did not receive any nutritional supplement beverage. For both supplemented groups, feeding with the assigned study product begun at 24–28 weeks gestation and continued for two months (minimum) after birth. Although optional, mothers were encouraged to continue for six months after birth. When the mother completely stopped breastfeeding, supplementation was also stopped. The infants continued in the study until one year of age.

**Fig. 1:**
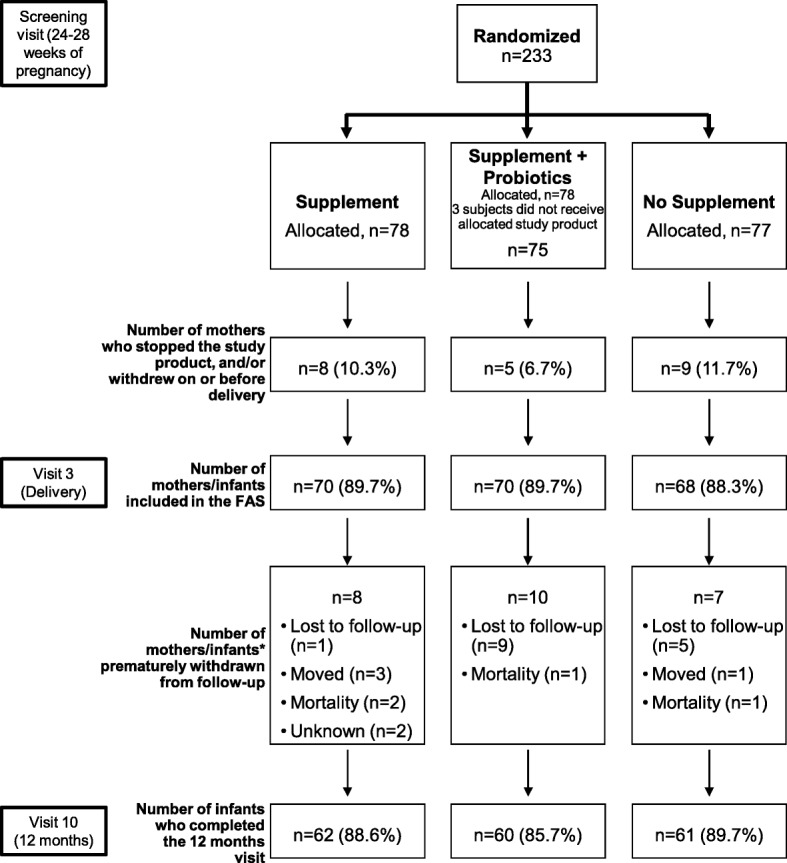
Disposition of study participants. FAS, full analysis set. *case report form did not distinguish between mothers/infants

A total of 11 visits (from screening visit 0 to last visit 10) took place during the study, the major visits are illustrated in Fig. 1. Before birth, expecting mothers attended a screening visit (at 24–28 weeks’ gestation, visit 0) during which history and subjects’ data were collected, inclusion/exclusion criteria were checked, health was assessed, and body weight measured. A fetal ultrasound was performed and women randomized to the supplemented groups started the supplements. At each visit, the physician inquired about the occurrence of AEs (especially nausea, vomiting, and diarrhea), assessed health, and measured body weight. At delivery (visit 3), the same information was recorded along with completion of a delivery record, including mode of delivery and gestational age. Mothers were followed and examined (for AEs and health assessments) up to 2 months post-delivery. At birth, infants were examined, anthropometric measurements were taken and AEs (any untoward birth defect) recorded. After birth, study investigators examined the infants at 2 weeks and 1, 2, 4, 6, 7, and 12 (visit 10) months of age and took anthropometric measurements, collected and reviewed parent/caregiver-reported 3-day feeding and gastrointestinal tolerance diaries, and assessed the occurrence of any AEs and in particular episodes and duration of diarrhea, since last visit.

### Outcome measures

The primary outcome was the incidence of diarrhea in infants from birth to one year (total count of diarrhea per infant per year). The incidence and duration of diarrhea episode were clinically assessed by the pediatrician at each visit, starting 2 weeks post-delivery through 12 months of age. Diarrhea was defined as at least three or more watery stools in a 24-h period. An episode of diarrhea was considered to have ended after two consecutive non-watery stools or no stools for 24 h.

In mothers, secondary outcomes included health assessment (clinical examination, weight gain); documentation of infections (genito-urinary tract infection); and tolerance to study product (nausea, vomiting, diarrhea).

Fetal growth (abdominal circumference, biparietal diameter, and femoral length) was determined by ultrasound at enrolment and just before delivery.

In infants, secondary outcomes included anthropometry and morbidity. Anthropometry was measured by weight, recumbent length/height and head circumference at each visit from birth to 12 months. Infants were weighed unclothed to the nearest 10 g on the same electronic scales calibrated according to the manufacturer’s instructions. Recumbent length was measured on a standardized length board to the nearest 1 mm with the body fully extended and feet flexed. Head circumference was measured using a standard plastic-coated measuring tape at approximately 2.5 cm above the eyebrows. Morbidity was inquired by the pediatrician at each visit and recorded in the AEs form.

### Adverse events and serious adverse events (SAEs)

The study investigator evaluated the seriousness of all AEs and any potential relation to the study products. AEs and SAEs were collected from randomization of the mothers up to the 12-month follow-up period, and coded using the WHO Adverse Reactions Terminology (WHO-ART). A serious AE was defined as any untoward medical occurrence that at any dose: results in death; is life-threatening; requires inpatient hospitalization or prolongation of existing hospitalization; results in persistent or significant disability/incapacity; or is a congenital anomaly/birth defect.

### Statistical methods

The sample size was calculated based on the articles of Hengstermann et al. [24] and Ortiz-Andrellucchi et al. [25]. Based on these two reference articles, a two sample test on proportion led to a number of 62 subjects per group in order to show a 50% decrease in incidence of diarrhea with a significance of 0.05 and a statistical power of 80%. Considering an estimated 20% of dropouts, a total number of 234 infants (78 per randomized group) were planned to be recruited into the study, and a total of 62 infants per group were supposed to be seen at 1 year of age. Those numbers were achieved as indicated in Fig. 1. The primary outcome was the total count of diarrhea per infant per year. The incidence of diarrhea was analyzed by Poisson regression in the full analysis set (FAS). Secondary outcomes (anthropometric measurements and other continuous outcomes) were analyzed by ANOVA correcting for sex and available baseline, respectively. A post-hoc analysis comparing the combined supplemented groups (oral nutritional supplements with and without probiotics) versus the no supplement group was performed. The aim of this post-hoc analysis was to assess the following outcomes in infants: body weight gain up to one year of age (non-inferiority analysis); anthropometric measurements at 12 months of age (body weight, length, head circumference and body mass index [BMI]); anthropometric WHO z-scores over time (weight-, length-, BMI- and head circumference-for-age); and infant’s morbidity. All outcomes were analyzed both by per protocol and by intention to treat data sets.

## Results

### Mothers and infants demographics and baseline characteristics

A total of 233 expecting mothers attending a single community hospital were randomized to one of the three study groups. Twenty-five women were not exposed to the study supplement and/or withdrew from the study on or before delivery. The results described in this section are based on the FAS population. Two hundred and eight mothers and the same number of infants were included in the FAS: 70 in the supplement group, 70 in the supplement plus probiotics group, and 68 in the no supplement group. Eight, 10, and 7 mothers/infants were excluded from the FAS in the supplement, supplement plus probiotics, and no supplement groups, respectively. A total of 183 infants completed the 12 months study visit (Fig. 1).

The demographics and baseline characteristics of mothers and infants were similar across the three groups (Table 2). All infants were in good health at birth with similar APGAR scores measured at 1 and 5 min after birth. The mean body weights at birth were similar for infants from the two supplemented groups (2.93 kg ±0.47 and 2.90 kg ±0.39, supplement and supplement plus probiotics, respectively) and for the no supplement group (2.88 kg ±0.44). At 4 months of age, 64.3% (supplement), 64.3% (supplement plus probiotics), and 72% (no supplement) of the infants were still on exclusive breastfeeding in their respective groups.

**Table 2:** Maternal and infants baseline characteristics

Characteristics	Supplement	Supplement + probiotics	No supplement
Mothers, n	**70**	**70**	**68**
Age at randomization in years, mean (SD)	26.3 (5.5)	24.8 (5.3)	24.9 (5.4)
Ethnic origin, % of Asian	100	100	100
First-time mothers, %	45.7	40.0	52.9
Type of delivery, % C-section	21.7	15.7	13.2
Education, % of secondary education	73.9	55.7	73.5
Infants at birth, n	**62**	**60**	**61**
Gestation age at birth in weeks, mean (SD)	38.8 (1.5)	38.8 (2.0)	38.8 (1.2)
Sex, % of males	50	62.9	44.1
APGAR score at 1 min, median (min-max)	9.0 (4.0–9.0)	9.0 (6.0–9.0)	9.0 (6.0–9.0)
APGAR score at 5 min, median (min-max)	9.0 (8.0–9.0)	9.0 (5.0–10.0)	9.0 (9.0–9.0)
Length in cm, mean (SD)	50.1 (2.7)	49.9 (2.8)	49.7 (2.4)
Weight in kg, mean (SD)	2.93 (0.47)	2.90 (0.39)	2.88 (0.44)
Body mass index in kg/m^2^, mean (SD)	11.6 (1.3)	11.6 (1.3)	11.6 (1.2)
Head circumference in cm, mean (SD)	32.7 (1.3)	32.8 (1.7)	32.5 (1.6)

*SD* Standard deviation

### Maternal supplementation during pregnancy

#### Maternal and fetal anthropometrics during last trimester of pregnancy

##### Maternal weight gain

Pre-pregnancy weight and BMI were very similar across all study groups, reflecting a homogeneous study population (Table 3). Despite a daily increase in calorie intake of about 280 kcal in the supplement and supplement plus probiotics groups, no significant difference in median maternal weight gain was observed between the supplemented groups and the no supplement group (supplement plus probiotics vs. no supplement, *p* = 0.945; supplement vs. no supplement, *p* = 0.892). Mean maternal weight gain at delivery was 10.6 kg, 10.5 kg, and 10.3 kg, in the supplement, supplement plus probiotics, and no supplement groups, respectively. Mean BMI at delivery was similar among groups (Table 3).

**Table 3:** Maternal weight status until delivery

Mean (SD)	Supplement (*n* = 70)	Supplement + probiotics (*n* = 70)	No supplement (*n* = 68)
Declared pre-pregnancy weight (kg)	49.1 (8.2)	49.2 (7.7)	49.1 (7.7)
Pre-pregnancy BMI (kg/m^2^)	20.6 (2.9)	20.7 (2.7)	21.1 (3.3)
Maternal weight gain from baseline (24–28 weeks of pregnancy) up to delivery (kg)	5.00 (2.69)	4.55 (2.25)	4.62 (2.16)
Maternal weight gain at delivery^a^ (kg)	10.6 (5.8)	10.5 (4.0)	10.3 (7.0)
Maternal BMI at delivery (kg/m^2^)	25.0 (3.2)	25.1 (3.0)	25.5 (3.4)

*BMI* body mass index; SD, Standard deviation. ^a^Calculated from participant report pre-pregnancy weight

##### Fetal growth and development

**Fig. 2:**
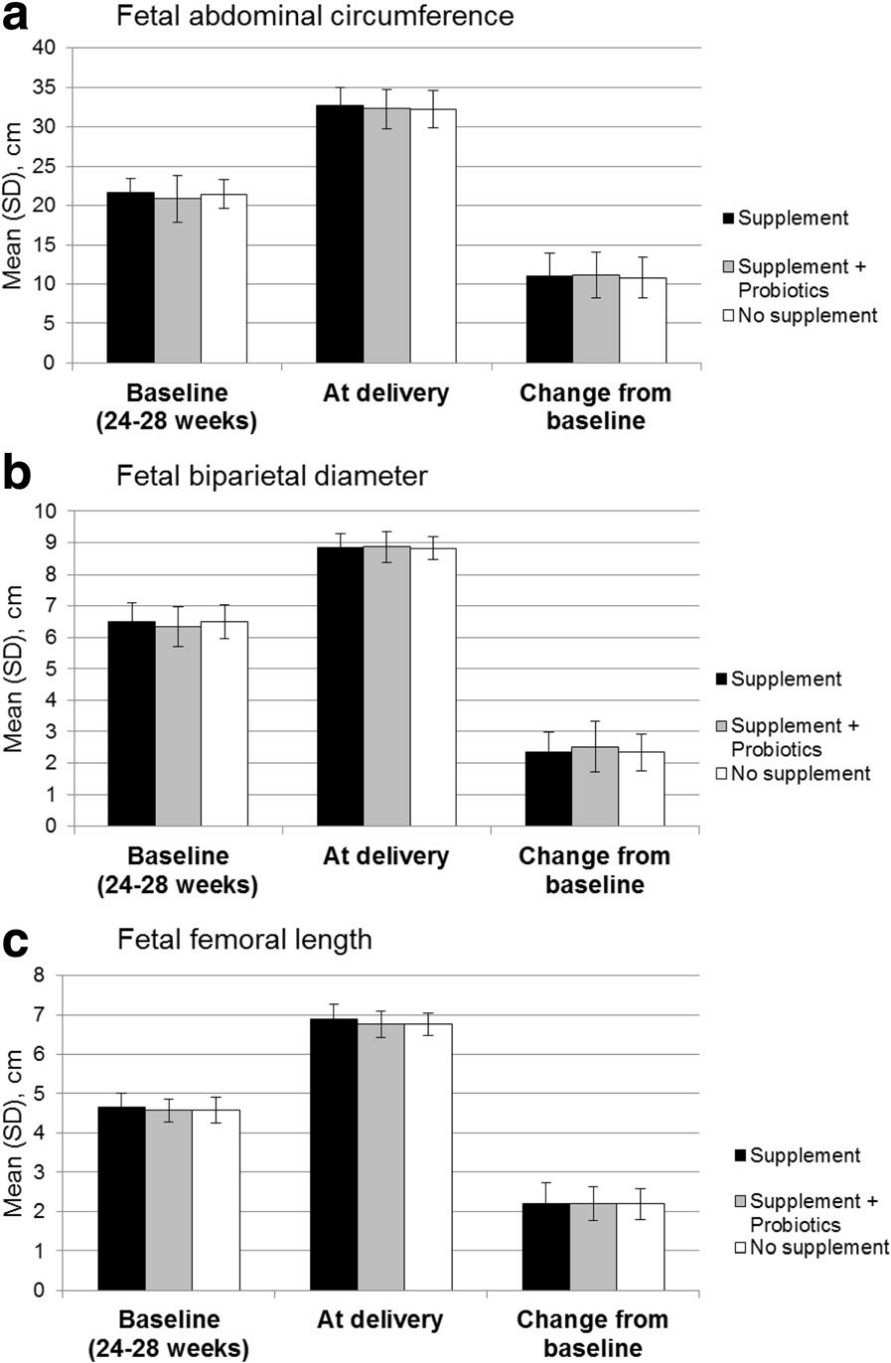
Fetal development. Fetal abdominal circumference (**a**), fetal biparietal diameter (**b**), and fetal femoral length (**c**)

Infants were born at approximately 39 weeks gestation with no statistically significant differences between the three groups in mean birth weight, APGAR scores, and anthropometrics (Table 2). Similarly, no significant differences were observed between the three groups for abdominal circumference (mean values of 32.64, 32.29, and 32.20 cm, in the supplement, supplement plus probiotics, and no supplement groups, respectively), biparietal diameter (mean values of 8.85, 8.86, and 8.82 cm in the supplement, supplement plus probiotics, and no supplement groups, respectively), or femoral length (mean values of 6.88, 6.75, and 6.76 cm, in the supplement, supplement plus probiotics, and no supplement groups, respectively) (Fig. 2). Overall, there were no statistically significant differences between the three groups for infant growth parameters during fetal development.

### Pregnancy-related AEs and SAEs

There were no statistically significant differences in the number of pregnancy-related AEs and SAEs recorded during the study between the three groups (Table 4). None of the SAEs were considered as related to the study supplements.

**Table 4:** Pregnancy-related serious adverse events (SAEs)

	Supplement (*n* = 70)		Supplement + probiotics (*n* = 70)		No supplement (*n* = 68)		
Pregnancy, puerperium and perinatal conditions	*# Events*	# Mothers (%)	*# Events*	# Mothers (%)	*# Events*	# Mothers (%)	p- value*
Fetal growth restriction	0	0 (0.0%)	0	0 (0.0%)	1	1 (1.5%)	*–*
Gestational hypertension	1	1 (1.4%)	1	1 (1.4%)	0	0 (0.0%)	*1.000*
Pre-eclampsia	1	1 (1.4%)	1	1 (1.4%)	1	1 (1.5%)	*1.000*
Premature labor	3	3 (4.3%)	1	1 (1.4%)	1	1 (1.5%)	*0.620*
Genito-urinary tract infections	15	14 (20.0%)	9	7 (10.0%)	3	3 (4.4%)	*0.154*
Tolerance: nausea, vomiting and diarrhea	6	4 (5.7%)	11	7 (10.0%)	1	1 (1.5%)	*0.532*

Data are number (%) of mothers with at least one event; *Supplement + probiotics versus Supplement

### Maternal supplementation during lactation

#### Infant outcome measures

##### Incidence of diarrhea

During the 12-month follow-up, 12.9% (9/70), 14.3% (10/70), and 16.2% (11/68) of infants experienced diarrhea in the supplement, supplement plus probiotics, and no supplement groups, respectively. One serious case of diarrhea was reported in the supplement group. There was no statistically significant difference in the incidence of diarrhea observed between the three groups. The incidence (95% confidence interval [CI]) was 0.17 (0.09–0.30) in the supplement group, 0.14 (0.07–0.27) in the supplement plus probiotics group, and 0.19 (0.11–0.33) in the no supplement group (supplement plus probiotics group vs. supplement group, *p* = 0.692; supplement plus probiotics group vs. no supplement group, *p* = 0.509). When supplemented groups were combined, the incidence (adjusted mean [95% CI]) was 0.15 (0.10–0.24) in the combined supplement groups vs. 0.19 (0.11–0.33) in the no supplement group (*p* = 0.586). The mean product effect with combined supplement was 0.82 (0.40–1.68) compared to no supplement.

##### Adverse events in infants

Overall, infant morbidity was similar among groups. However, there was a trend towards more serious gastrointestinal infections in the no supplement group (10.3%) than in the combined supplemented groups (4.3%). Moreover, we noticed significantly less frequent vomiting cases in the combined supplemented group compared to no supplement group (0.0% vs. 5.9%, *p* = 0.011). In total, there were four infant deaths. Two deaths were reported in the supplement group, one in the supplement plus probiotics group, and one in the no supplement group (Table 5). None of these cases were considered related to product consumption in the medical records.

**Table 5:** Adverse events in infants (combined supplemented groups versus non-supplemented group)

	Combined^§^ (*n* = 140)		No Supplement (*n* = 68)		*p-* value* Combined vs. No supplement
	*# Events*	# Infants (%)	*# Events*	# Infants (%)	
Adverse events of diarrhea					
Serious diarrhea	1**	1 (0.7%)	0	0 (0.0%)	*1.000*
Diarrhea	21	19 (13.6%)	12	11 (16.2%)	*0.675*
*Total*	22	20	12	11	*–*
Other serious adverse events of interest					
All respiratory infections/symptoms	309	119 (85.0%)	177	63 (92.6%)	*0.179*
Gastrointestinal infections	7	6 (4.3%)	7	7 (10.3%)	*0.126*
Allergies	11	7 (5.0%)	4	3 (4.4%)	*1.000*
Death^a^	3	3 (2.4%)	1	1 (1,5%)	
Other adverse events of interest					
Vomiting	0	0 (0.0%)	5	4 (5.9%)	*0.011*

*Data are number of infants with at least one event (%).*^§^*Combined:* supplement and supplement + probiotics groups.*Exact Fisher test p-value.**Event reported in the supplement group. ^*a*^*As confirmed by clinicians, not related to product consumption*

##### Changes in anthropometric measurements over time

**Fig. 3:**
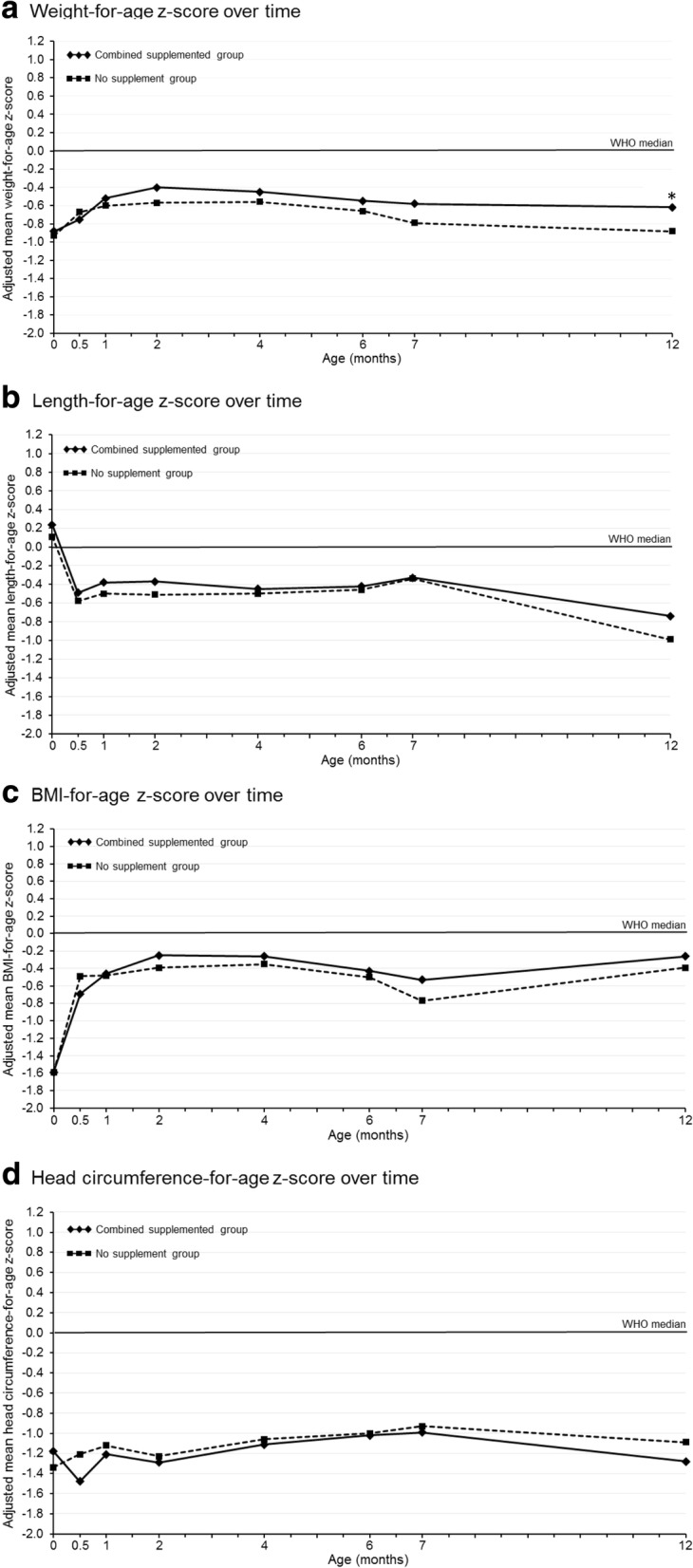
Infant anthropometrics over the study period. Weight-for-age (**a**), length-for-age (**b**), BMI-for-age (**c**) and head circumference-for-age (**d**) z-scores (World Health Organization growth reference). *p = 0.045

In the three study groups, mean weight-for-age, length-for-age, BMI-for-age and head circumference-for-age z-scores were below the WHO median values. At birth, all anthropometric measurements were similar between the groups. Interestingly, at 2 months of age, i.e. at the end of the mandatory intake of maternal supplementation and breastfeeding, the combined supplemented group had higher weight-for-age (− 0.40, 95% CI: -0.54 to − 0.25 vs. -0.57, 95% CI: -0.77 to − 0.36), length-for-age (− 0.37, 95% CI: -0.55 to − 0.19 vs. -0.51, 95% CI: -0.77 to − 0.25), and BMI-for-age (− 0.25, 95% CI: -0.43 to − 0.07 vs. -0.39, 95% CI: -0.65 to − 0.14) z-scores than the no supplement group (Fig. 3a, b, c, ). However, these differences did not reach significance. This trend persisted throughout the study follow-up period, up to 12 months. At 12 months of age, the combined supplemented groups gained more weight (adjusted mean in kg, 95% CI: 8.97 [8.84–9.09] vs. 8.61 [8.43–8.78]; *p* = 0.001) and height (adjusted mean in cm, 95% CI: 74.2 [73.8–74.6] vs. 73.4 [72.8–74.0]; *p* = 0.031) compared to the no supplement group (Table 6) and had a significantly higher weight-for-age z-score (− 0.88 vs. -0.62, *p* = 0.045) (Fig. 3a). There was neither statistically significant difference nor trend for a higher head circumference-for-age z-score in the combined versus no supplement groups throughout the study (Fig. 3d). No statistically significant differences on growth outcome per gender were observed between groups (data not shown).

**Table 6:** Infant anthropometrics at 12 months of age

Measurement, adjusted mean (95% CI)	*Supplement (n = 62)*	Supplement + probiotics (*n* = 60)	Combined^a^ (*n* = 122)	No supplement (*n* = 61)	*p-value*	
					Supplement + probiotics vs. supplement	Combined vs. No supplement
Weight (kg)	8.92 (8.75–9.09)	9.06 (8.88–9.24)	8.97 (8.84–9.09)	8.61 (8.43–8.78)	*0.273*	*0.001*
Length (cm)	73.8 (73.3–74.4)	74.6 (74.1–75.2)	74.2 (73.8–74.6)	73.4 (72.8–74.0)	*0.057*	*0.031*
Head circumference (cm)	44.0 (43.7–44.3)	44.1 (43.9–44.5)	44.1 (43.9–44.3)	44.2 (44.0–44.7)	*0.413*	*0.317*
Body mass index (kg/m^2^)	16.3 (16.0–16.7)	16.1 (15.8–16.5)	16.2 (16.0–16.5)	16.0 (15.7–16.4)	*0.496*	*0.371*

Analyzed by ANCOVA, correcting for sex and available baseline; ^a^Combined: supplement and supplement + probiotics groups; *CI* confidence interval

## Discussion

In the present study, we assessed the effect of an oral maternal nutritional supplement formulated with the probiotics *Lactobacillus rhamnosus* and *Bifidobacterium lactis* on the incidence of diarrhea in infants from birth to one year. The supplement was consumed by expecting mothers during the third trimester of pregnancy and continued for at least two months post-partum while breastfeeding. There was no significant difference in the incidence of infant diarrhea between treatment groups. The incidence of diarrhea was also similar between the supplemented groups and the no supplement group. In a clinical trial setting, an overall lower incidence of infant diarrhea is often observed as compared to field data records. It is likely that mothers involved in clinical trials provide more care and attention to their infants than during everyday life when infants do not undergo such a constant growth and health monitoring. Nevertheless, in the present study the incidence rate of infant diarrhea was not dramatically lower than the estimates from the two reference articles used for sample size calculation [24, 25]. Even if the supplement plus probiotics group pointed towards a slight numerical trend compared to the supplement and no supplement groups, the expected 50% reduction in incidence of infant diarrhea was not achieved in the current clinical trial setting. In order to reach statistical significance more than 300 infants per group would have been required. In this context, one could consider that the study was underpowered to show such a dramatic impact on incidence of infant diarrhea.

Secondary endpoints were evaluated as planned, and an additional post-hoc analysis was performed. It compared the combined groups receiving oral supplements (with and without probiotics) with the no supplement group to evaluate the effect of maternal supplementation on maternal health and fetal and infant growth. Some studies have described the safety and effect of a nutritional supplement containing a mix of probiotics for pregnant and breastfeeding women on fetus and infant [12–15], but none have fully examined the impact of oral nutritional supplements on fetal and infant growth. Our study provides preliminary evidence suggesting a beneficial effect of oral nutritional supplements, with and without probiotics, during the third trimester of pregnancy on optimal maternal weight gain and infant growth at one year.

Oral maternal nutritional supplements are formulated to provide a balance of energy, protein and nutrients to support a healthy pregnancy. However the consumption of too many calories during pregnancy may increase a woman’s risk for excessive weight gain which has been linked with gestational diabetes [26, 27] and hypertension [26], cesarean birth [26, 28]**,** macrosomia [28–30] and large-for-gestational age infants [26, 28], later type 2 diabetes [31] and obesity [26], along with childhood obesity [26]. During the third trimester of pregnancy, our study showed that maternal weight gain among the three study groups was equivalent despite an increase in calorie intake of 280 kcal per day in the supplemented groups. This is consistent with a recent report indicating a reduced occurrence of central adiposity (i.e. waist circumference ≥ 80 cm) at 6 months after birth when probiotics are given to pregnant women in combination with dietary counseling [32]. Moreover, our results are in agreement with the findings of Luoto et al. who reported a reduction of gestational diabetes in mothers fed probiotics *Lactobacillus rhamnosus* and *Bifidobacterium lactis* together with dietary counseling from the first trimester of pregnancy and adequate prenatal and postnatal growth rates (body weight and length) of infants during the first two years of life [33]. Additionally, our study showed no differences in the incidence of pregnancy-related AEs and SAEs, and adverse fetal outcomes between all study groups. Overall, the optimal maternal weight gain and positive tolerance data indicate that maternal feeding with a supplement containing or not probiotics during the last trimester of pregnancy is safe for the mother and the fetus with respect to its energy density and nutritive value.

We further explored the effect of the supplementation compared to the current nutrition practice, i.e. no supplement. The two supplemented groups were thus subsequently pooled and compared to the no supplement group in a *post-hoc* analysis. No difference was observed between the two groups with respect to AEs or SAEs in infants, except for vomiting which appeared significantly less frequent in the combined supplemented group than in the no supplement group. Moreover, a trend for less serious gastrointestinal infections in the combined supplemented group was noticed. Altogether, these observations are suggestive of the potential beneficial effect of the maternal supplement on infant health, regardless of the addition of probiotics. Additional studies would be needed to confirm these observations.

As giving nutritional supplements to breastfeeding mothers in developing countries could presumably improve infant growth, in the *post-hoc* analysis, we also examined the effect of the combined supplemented group on infant growth. In our study, all infant growth parameters were below WHO median values, at all study time points (up to 12 months of age) and in both the combined supplemented and the no supplement groups. This finding is consistent with the results from the Food and Nutrition Research Institute of the Department of Science and Technology, 8th National Nutrition Survey showing an estimated 19.9% of Filipino children aged 0 to 5 years are underweight [34]. Nevertheless, it should be noted that the growth curves of the combined supplemented group was higher (except for head circumference) compared with the no supplement group for all time points.

Starting around 6–7 months of age, a negative break in the infant growth curves was observed which probably corresponds to the start of the weaning period. At this time, complementary foods are introduced and the infant is at an increased risk of being exposed to food-borne illness. Furthermore, complementary foods may often be of lower nutritional value than infant formula, resulting in the consumption of a non-optimal amount of calories, protein and essential micronutrients. In the Philippines, among the lower socio economic population, an increased risk of infection combined with inadequate nutrition may be contributing to the decline in infant growth rate. This could explain what was observed at 6 months of age.

At 12 months of age, infants in the combined supplemented group had gained significantly more weight than the infants in the no supplement group. This suggests a potential long-term beneficial effect of the supplementation. The GUSTO (Growing Up in Singapore Towards healthy Outcomes) study group recently showed that food consumption was altered during pregnancy and the postpartum period in a large number of women from a multiethnic Asian population who were heavily influenced by traditional beliefs surrounding these periods [35]. This study group further investigated the association between maternal macronutrient intake during pregnancy and infant birth size in the same Asian population [36]. Similar to what was observed in the no supplement group of our study, the study concluded that maternal macronutrient intake was not associated with infant birth weight [36]. Additionally, Bhargava examined the effect of dietary intake on growth and morbidity in Filipino infants from 2 to 24 months and reported calcium, protein to energy intake ratio, and β-carotene were significantly correlated with height, weight, and morbidity indexes, respectively [37]. In western infants, clinically significant catch-up growth (defined as a gain in weight z-scores > 0.67) between 0 and 24 months was associated with greater weight, height, BMI and body fat at five years compared to similar aged children without catch-up growth [38]. In this study, the mean product effect on weight-for-age z-scores was only equal to 0.211. Nevertheless, there is a need for a better understanding of the benefits and risks of greater weight gain to promote improved health outcomes and the later risk of adiposity and obesity [39]. Further investigating of these potential determinants of excess infant adiposity gain could lead to intervention strategies in clinical and public health settings to prevent childhood obesity and its consequences.

We acknowledge some limitations in our study. First, supplement consumption was not monitored beyond the 2-month postnatal period, and the impact of continued supplement consumption on breast-fed infant growth was not examined. Second, the potential beneficial effect of the supplements on breast milk composition was not studied and compared between groups. This could have provided insights on the effect observed on infant growth. Third, the absence of increased weight gain in pregnant women taking the supplement (with or without probiotics) cannot be generalized to the whole population as this observation may be linked to a genetic component specific to this sample of Filipino women. Indeed, women’s weight in Western countries is generally higher and women tend to gain more weight during pregnancy (for women with a normal weight, i.e. a BMI of 18.5–24.9, the recommended range of total weight gain during pregnancy is 11–16 kg [40]). Moreover, the mother’s diet and exercise during the third trimester of pregnancy and the 2-month postnatal period was not taken into account, as these data were not recorded during the trial. However, considering that all recruited mothers had comparable socioeconomic profiles and came from the same geographical area, one can assume that their global calorie intake was similar. Nevertheless, a better monitoring of mothers’ diet and exercise should be included in future nutritional intervention trials. Finally, limitations also include the study population of women in the third trimester of pregnancy living in the greater Manila region, limiting the generalizability of these findings to all stages of pregnancy and all regions of the Philippines.

## Conclusion

Oral maternal nutritional supplements formulated with or without *Lactobacillus rhamnosus* and *Bifidobacterium lactis* during the last trimester of pregnancy and through the first two months of exclusive breastfeeding were well tolerated and safe for mothers, fetus and infants. Even though in the present study no difference in incidence of infant diarrhea was observed between the three groups, the analysis of combined supplemented groups showed beneficial effects of maternal supplementation on infant weight and length gains at 12 months of age.

## Abbreviations

AEs: Adverse events
BMI: Body mass index
CFU: Colony forming units
CI: Confidence interval
FAS: Full analysis set
GI: Gastro-intestinal
HIV: human immunodeficiency virus
SAEs: Serious adverse events
SD: Standard deviations
WHO: World Health Organization
WHO-ART: WHO adverse reactions terminology
